# Investigating the influence of PFC transection and nicotine on dynamics of AMPA and NMDA receptors of VTA dopaminergic neurons

**DOI:** 10.1186/1743-0003-8-58

**Published:** 2011-10-21

**Authors:** Ting Chen, Die Zhang, Andrei Dragomir, Kunikazu Kobayashi, Yasemin Akay, Metin Akay

**Affiliations:** 1 Department of Biomedical Engineering, Cullen College of Engineering, University of Houston, Houston, TX 77204, USA; 2 Division of Computer Science and Systems Engineering, Graduate School of Science and Engineering, Yamaguchi University, Ube, Yamaguchi 755-8611, Japan

## Abstract

**Background:**

All drugs of abuse, including nicotine, activate the mesocorticolimbic system that plays critical roles in nicotine reward and reinforcement development and triggers glutamatergic synaptic plasticity on the dopamine (DA) neurons in the ventral tegmental area (VTA). The addictive behavior and firing pattern of the VTA DA neurons are thought to be controlled by the glutamatergic synaptic input from prefrontal cortex (PFC). Interrupted functional input from PFC to VTA was shown to decrease the effects of the drug on the addiction process. Nicotine treatment could enhance the AMPA/NMDA ratio in VTA DA neurons, which is thought as a common addiction mechanism. In this study, we investigate whether or not the lack of glutamate transmission from PFC to VTA could make any change in the effects of nicotine.

**Methods:**

We used the traditional AMPA/NMDA peak ratio, AMPA/NMDA area ratio, and KL (Kullback-Leibler) divergence analysis method for the present study.

**Results:**

Our results using AMPA/NMDA peak ratio showed insignificant difference between PFC intact and transected and treated with saline. However, using AMPA/NMDA area ratio and KL divergence method, we observed a significant difference when PFC is interrupted with saline treatment. One possible reason for the significant effect that the PFC transection has on the synaptic responses (as indicated by the AMPA/NMDA area ratio and KL divergence) may be the loss of glutamatergic inputs. The glutamatergic input is one of the most important factors that contribute to the peak ratio level.

**Conclusions:**

Our results suggested that even within one hour after a single nicotine injection, the peak ratio of AMPA/NMDA on VTA DA neurons could be enhanced.

## Background

Nicotine is thought to be the biologically active substance that promotes tobacco use. Approximately a quarter of the global population uses tobacco products that cause health and economical problems. Unfortunately, nicotine dependence creates problems for smokers to quit. The dopamine (DA) neurons in the ventral tegmental area (VTA) and their projection areas, including prefrontal cortex (PFC), nucleus accumbens (NAc), and amygdala, are thought to be very important in the reward-driven behavior-induced process by the drugs of addiction [1-5].

Malenka et al established a model to assess the glutamate receptor (GluR) plasticity and altered synaptic function by examining *in vitro *VTA DA neurons from midbrain slice preparation following 24 hours of a single, systemic administration of several types of drugs of addiction [6,7]. Following administration, they found that the peak ratio of α-amino-3-hydroxy-5-methyl-4-isoxazole propionic acid (AMPA) receptor-mediated excitatory postsynaptic currents (EPSCs) to N-methyl-D-aspartate (NMDA) receptor-mediated EPSCs was enhanced, which reflects a glutamatergic synapse plastic alteration onto DA neurons in the VTA. This may underlie a common mechanism of neural adaptation to addictive drugs [7].

Previous reports have shown that DA is released to NAc and locomotion activity *in vivo *has peaked with nicotine injection within one hour [8]. Additionally, long term potentiation (LTP) was rapidly induced by afferent stimulation and lasted more than one hour in VTA slice [9]. Moreover, a positive correlation between glutamatergic synaptic enhancement and behavioral locomotion existed [10].

The firing activities of VTA DA neurons and addictive behavior of the animals are believed to be controlled, impartially, by the glutamatergic synaptic inputs from PFC [11-15]. Evidence has shown that, the functional input loss from PFC and/or NAc may reduce the effects of these drugs on the addiction process [14,16-18]. In VTA, the AMPA/NMDA receptors' ratio response of dopamine neurons was found to be enhanced only by the drugs of abuse, and the enhanced ratio was thought to be caused by the excitatory input increase, which mostly originate from PFC [6,7].

Previous studies showed that, the strengthening of input from PFC to VTA plays an important role in the development of behavioral sensitization, a well-known model for addiction [7,19-22]. We recently showed that in *in vivo *experiments, acute response of VTA to nicotine with PFC transection is significantly changed when compared to PFC intact subjects [23,24].

Thus far, it is still unknown how the AMPA/NMDA peak ratio changes without PFC projection. In our study, we investigate whether the synaptic strength would increase following only one hour after single nicotine administration by activating multiple molecular and cellular cascades.

In addition to the AMPA/NMDA peak ratio measurement proposed by the other research groups, we performed analysis of the synaptic response by estimating the areas under the AMPA and NMDA EPSC waveforms [25,26]. This allows us to better understand the dynamics of the synaptic charge transfer. Moreover, we used the Kullback-Leibler (KL) divergence analysis method to quantitatively evaluate the difference between the shapes of the AMPA and NMDA signals [27].

## Methods

### Animals and treatment

We used Sprague Dawley rats (14-19 days old) for the experiments [9]. All experimental protocols and surgeries were approved by The Institutional Animal Care and Use Committee of Arizona State University. For PFC intact animals, the skin on the skull was cut to mock the surgery under anesthesia (isofluran USP) and was sutured after the manipulation. The subjects were given one hour to recover from the anesthesia effect before saline (volume matched to nicotine injection) or nicotine (0.5 mg/kg) was intraperitoneal (i.p.) injected.

The subjects in the PFC transected group were under anesthesia (isofluran USP) while bilateral transections were made immediate caudal to the PFC to disrupt the connection between PFC and VTA DA neuron with the skin on the head open. A slit was drilled in the skull around 1 mm anterior to bregma. A sharp blade was lowered to the base of skull, without damaging the main artery, to completely interrupt the connections between the PFC and the rest of the brain [28]. The post-surgical care and drug administration were identical to the PFC intact animals.

The disruption between PFC and VTA was observed at the time the brain was removed from the skull. Once the brain has been removed from the skull, the area immediate caudal to PFC has been observed to be cut. This indicates the PFC has completely lost its connection with the rest of the brain.

### Electrophysiological recordings

One hour after single systemic injection of nicotine, animals were anesthetized by forane (isoflurane USP) and sacrificed. The remaining procedures were identical as previously described [6]. Briefly, horizontal midbrain slices (250 μm) were cut using a vibratome 1000 (Vibratom, St. Louis, MO). Slices were prepared in ice-cold artificial cerebrospinal fluid (ASCF) solution containing (in mM): 126 NaCl, 1.6 KCl, 1.2 NaH_2_PO_4_, 1.2 MgCl_2_, 2.5 CaCl_2 _, 18 NaHCO_3 _and 11 glucose. The slices were incubated for at least one hour in a holding chamber at room temperature (22-24°C) and continuously bubbled with 95% O_2 _and 5% CO_2 _carbogen in the same ACSF solution. Conventional whole-cell recordings were made using a patch clamp amplifier (Multiclamp 700B, Axon Instruments) under infrared-DIC microscopy (Axioskop2 FS Plus, Zeiss). Data acquisition and analysis were performed using a digitizer (DigiData 1440A, Axon Instruments) and the analysis software pClamp 10.2 (Axon Instruments). Signals were filtered at 2 kHz and sampled at 10 kHz. For presynaptic stimulation, a bipolar tungsten stimulation electrode (WPI, Sarasota, Florida) was placed 100-200 μm rostral to the recording electrode to stimulate excitatory afferents, stimulation pulse of 40 μs duration and 0.1 Hz frequency were applied. For measurements of the ratio of AMPA and NMDA receptor-mediated currents, the DA neuron was voltage-clamped at +40 mV. Picrotoxin (100 μM) was added to the bath solution to block GABA_A_-receptor-mediated inhibitory synaptic transmission. Initially, a stable baseline recording of total evoked EPSCs was obtained for 5 min. Then the NMDA receptor antagonist AP-V (50 μM) was applied to the bath for 10 min to obtain AMPA-receptor-mediated EPSCs. An average of 15 evoked EPSCs was collected for each type of EPSC. NMDA-receptor-EPSCs were obtained by digitally subtracting the AMPA-receptor-EPSCs from the total EPSCs from the same neuron. For the ratio experiments, the whole-cell recording pipette (3-6 MΩ) was filled with a solution containing (in mM): 117 cesium methansulfonic acid, 20 HEPES, 0.4 EGTA, 2.8 NaCl, 5 TEA-Cl, 2.5 MgATP and 0.25 GTP (pH 7.2-7.4 with CsOH). Series resistance was monitored throughout the whole-cell recording. Only two slices were obtained from each animal and a single cell was examined from each slice. All values are expressed as mean ± SEM. Statistical significance was assessed using two-tailed Student's t- tests.

**Figure 1:**
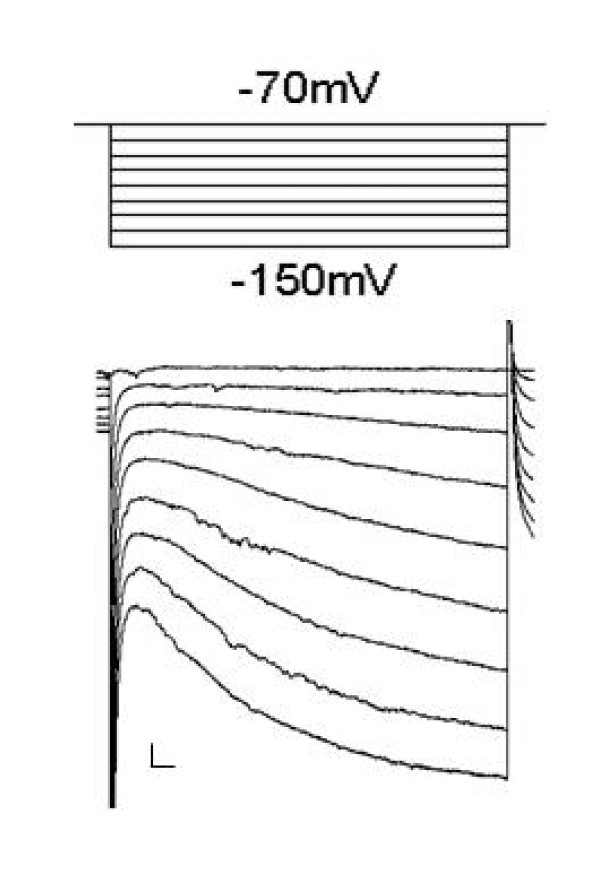
**Example of Ih currents were observed for midbrain DA neurons under voltage clamp**. The holding potential was -70 mV and was given -10 mV step size to reach -150 mV (calibration bars: 20 pA/50 ms).

All recordings were performed at 31 ± 1°C [6,7]. The DA neuron was identified by large hyperpolarization-activated current (I_h_) as shown in Figure 1[29,30]. All drugs were obtained from Sigma, unless otherwise specified.

### AMPA/NMDA area ratio

The area under AMPA and NMDA EPSC curves was estimated. This area represents the synaptic charge transfer [25,26]. For each pair AMPA/NMDA of each VTA DA neuron the area ratios were computed for 50 ms length segments along the signal. This approach allows us to estimate the way charge transfer dynamically evolves along the synaptic response. At the same time it enables us to compare the changes brought to the synaptic charge transfer by different experimental conditions (PFC intact vs transected; saline treated vs nicotine treated).

### Kullback-Leibler divergence

The Kullback-Leibler (KL) divergence method originates from information theory and is a quantitative measure of the difference between two probability distributions [27]. The KL divergence of distribution *q*(*x*) from distribution *p*(*x*), *KL*(*p*||*q*), is mathematically defined as:

(1) $$KL\left(p||q\right)=\int p\left(x\right)\;\;\ln\frac{p\left(x\right)}{q\left(x\right)}dx.$$

From its properties, the KL divergence satisfies *KL*(*p*||*q*) ≥ 0 with equality if and only if *p*(*x*) = *q*(*x*), and is asymmetric quantity, i.e. *KL*(*p*||*q*) ≠ *KL*(*q*||*p*).

In this analysis, we used the following measure,*KL*(*p*, *q*), as a KL divergence to treat it as symmetric quantity [31].

(2) $$\begin{array}{lll} KL\left(p,q\right) & =KL\left(p||q\right)+KL\left(q||p\right)\; & \text{(1)}\\ & =\int \left(p\left(x\right)-q\left(x\right)\right)\;\;\ln\frac{p\left(x\right)}{q\left(x\right)}dx.\; & \text{(2)}\\ & \; & \text{(3)} \end{array}$$

In information theory, *p*(*x*) and *q*(*x*) are assumed as probability distributions. In the present study, however, we assumed that *p*(*x*) and *q*(*x*) correspond to AMPA and NMDA receptor-mediated EPSCs, respectively. Under this assumption, we can quantitatively evaluate the difference between the shapes of the AMPA and NMDA signals using the KL divergence. This measure provides information on the whole area of synaptic response and not just the maximum response value, as a measure based on the peak ratio would.

Since all the recording data of AMPA and NMDA signals is sampled and has discrete values, we need to transform the KL divergence in Eq.(2) to a discrete format as below:

(3) $$KL\left(p,q\right)=\overset{N}{\underset{i=1}{\sum }}\left(p\left(x_{i}\right)-q\left(x_{i}\right)\right)\;\;\ln\frac{p\left(x_{i}\right)}{q\left(x_{i}\right)},$$

where *x_i_*means an *i *-th discrete signal and *N *is the number of recording data. Before calculating the KL divergence using Eq.(3), we need to preprocess the signals to take positive values and their sums equal to one, i.e. $\overset{N}{\underset{i=1}{\sum }}p\left(x_{i}\right)\;=\overset{N}{\underset{i=1}{\sum }}q\left(x_{i}\right)\;=1$ because of an original restriction on the probability distribution in information theory.

We calculated the KL divergence for each pair of AMPA and NMDA signals, under different experimental conditions (nicotine and saline and also with PFC intact and transected rats). Subsequently, the statistical significance of the difference between AMPA and NMDA signals under the different conditions was assessed using two-tailed Student's t-tests.

## Results

### AMPA/NMDA peak ratio

**Figure 2:**
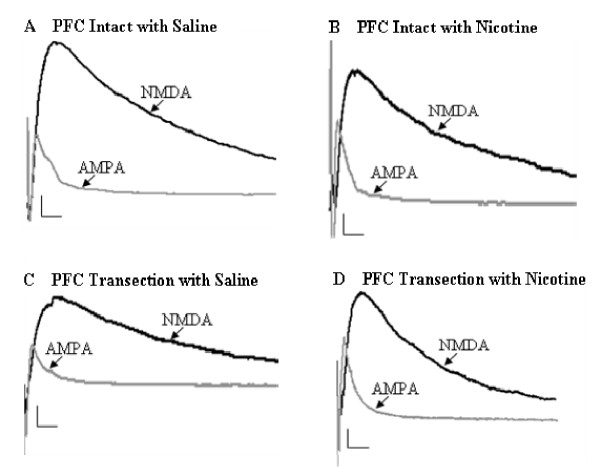
**Example recordings of evoked NMDA and AMPA EPSCs from midbrain VTA DA neurons of rats one hour after treatment of saline and nicotine with PFC intact and PFC transection (calibration bars: 20 pA/15 ms)**.

**Figure 3:**
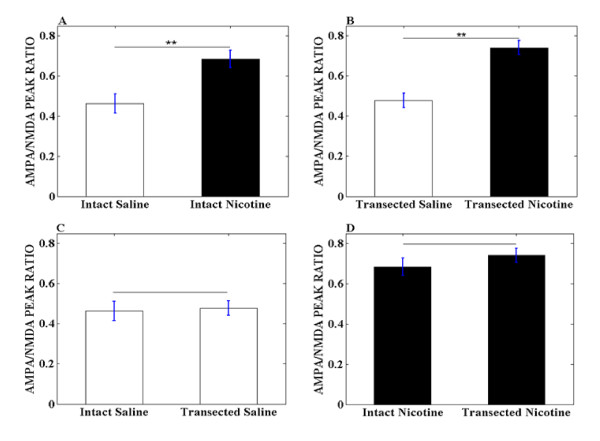
**Summary of AMPA/NMDA peak ratios obtained from rats with different treatments**. (A) Summary of AMPA/NMDA peak ratio obtained from rats treated with saline and nicotine with PFC intact (** indicates p < 0.01). (B) Summary of AMPA/NMDA peak ratio obtained from rats treated with saline and nicotine with PFC transected (** indicates p < 0.01). (C) Summary of AMPA/NMDA peak ratio obtained from rats treated with saline with PFC intact and PFC transected. (D) Summary of AMPA/NMDA peak ratio obtained from rats treated with nicotine with PFC intact and PFC transacted.

The measurement of glutamatergic synaptic strength was applied exactly as previously described [6,7], in which, the AMPA receptor-mediated EPSCs was normalized to NMDA receptor-mediated EPSCs to obtain the peak ratio of AMPA/NMDA as seen in Figure 2. In the nicotine treated group, one hour after single injection of nicotine with PFC intact, the AMPA/NMDA peak ratio was 0.68 ± 0.04 (n = 6), while in saline group, that was 0.46 ± 0.035 (n = 7) as seen in Figure 3A. This significant enhancement induced by nicotine treatment (p < 0.01) is consistent with another previous report that 24 hours after a single, systemic administration of nicotine enhances the excitatory synapse strength on VTA DA neurons by enhancement of postsynaptic AMPA receptors [7].

To investigate whether PFC transection would cause the AMPA/NMDA peak ratio to be different, we repeated the same experiments in PFC transection rats. In response to the PFC transection, the saline group has peak ratio of 0.48 ± 0.035 (n = 7), while the nicotine group exhibited 0.74 ± 0.035 (n = 7). The results show that nicotine treatment still could increase the AMPA/NMDA peak ratio significantly (p < 0.01), even without intact inputs from PFC as seen in Figure 3B.

After confirming the nicotine's enhancing effects in both PFC intact and PFC transection rats, we investigated whether there is any difference in the effects of nicotine between these two groups. As showed in Figure 3D, the peak ratio for PFC intact with nicotine is 0.68 ± 0.04 (n = 6), while PFC transection with nicotine is increased to 0.74 ± 0.035 (n = 7). However, these changes are not significant.

### AMPA/NMDA area ratio

**Figure 4:**
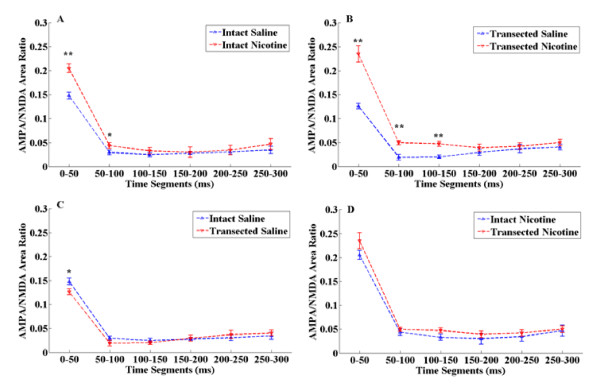
**Summary of AMPA/NMDA area ratio obtained from rats with different treatments**. (A) Summary of AMPA/NMDA area ratio obtained from rats treated with saline and nicotine with PFC intact (** indicates p < 0.01, * indicates p < 0.05). (B) Summary of AMPA/NMDA area ratio obtained from rats treated with saline and nicotine with PFC transected (** indicates p < 0.01). (C) Summary of AMPA/NMDA area ratio obtained from rats treated with saline with PFC intact and PFC transected (* indicates p < 0.05). (D) Summary of AMPA/NMDA area ratio obtained from rats treated with nicotine with PFC intact and PFC transacted.

We also performed analysis of the synaptic response by estimating the areas under the AMPA and NMDA EPSC curves. This allows us to better understand the dynamics of the synaptic charge transfer and provides more information than traditional measures based on only peak ratios. Specifically, as mentioned in the method section, we estimated areas under AMPA and NMDA curves on consecutive 50 ms segments. For each segment the AMPA/NMDA area ratio was computed. From Figure 4A, we observe that nicotine treatment induced a significant difference (p < 0.01) on the synaptic charge and hence on the AMPA/NMDA area ratio, when compared to saline on the first 50 ms of the synaptic response. The difference continues to be significant (p < 0.05) up to 100 ms, while subsequently, the synaptic charge transfer seems to be unaffected by nicotine.

A similar trend is also apparent when comparing the effect of nicotine treatment after PFC transection occurred. However, in this case the transection seems to prolong the differences in the synaptic charge induced by nicotine, when compared to saline. As observed in Figure 4B, the area ratios are significantly different up to 150 ms (p < 0.01).

The area ratio measure offered a substantial different view than the peak ratio when comparing the saline treated PFC intact and PFC transected responses. When using the peak ratio method, we could not identify any difference in the synaptic responses. However, the area ratio shows us there is a significant effect induced by PFC transection on the first 50 ms (p < 0.05) of saline treated group as seen in Figure 4C.

### KL Divergence

**Figure 5:**
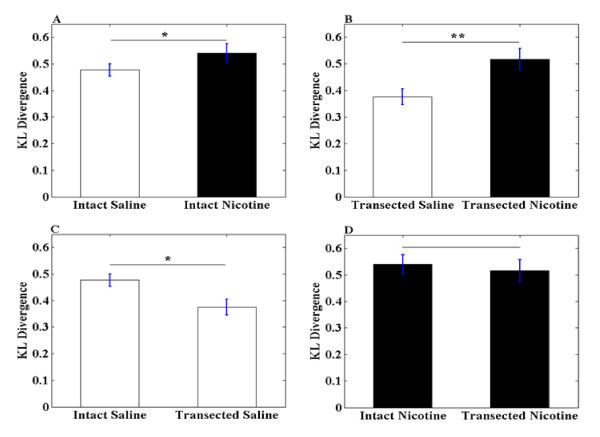
**Summary of KL divergence obtained from rats with different treatments**. (A) Summary of KL divergence obtained from rats treated with saline and nicotine with PFC intact (* indicates p < 0.05). (B) Summary of KL divergence obtained from rats treated with saline and nicotine with PFC transected (** indicates p < 0.01). (C) Summary of KL divergence obtained from rats treated with saline with PFC intact and PFC transected (* indicates p < 0.05). (D)Summary of KL divergence obtained from rats treated with nicotine with PFC intact and PFC transacted.

Additionally, we estimated the KL divergence for signals treated with nicotine and saline and also with PFC intact and transected rats throughout the whole current response. The EPSCs are assumed as probability distribution. The advantages of the analysis based on KL divergence arise from the higher level information provided, since it allows us to estimate differences in the shapes of the AMPA and NMDA currents. From Figure 5A, one hour after single injection of nicotine for PFC intact rats, the KL divergence was 0.54 ± 0.036, while in saline group, that was 0.48 ± 0.024. We observed there is significant difference (p < 0.05) between nicotine treated and saline treated PFC intact group.

In Figure 5B, the saline group has KL divergence of 0.38 ± 0.030, while the nicotine group exhibited 0.52 ± 0.041. The results show that nicotine treatment still could increase the KL divergence significantly (p < 0.01), even without intact inputs from PFC.

However, the KL divergence analysis measure also offered a significant different view than the peak ratio when the saline treated PFC intact and PFC transected responses was compared. As seen in Figure 5C, results using KL divergence analysis method showed there is a significant difference (p < 0.05) with PFC transection that is not observed using the peak ratio method.

It is worth noting that, with PFC intact and PFC transected saline treatment, there is significant difference in the responses as seen in Figures 4C and 5C. Whereas PFC intact and PFC transected nicotine treatment has no significant difference. This result led us to believe that with PFC transection, the VTA DA neurons are more sensitive to nicotine exposure.

## Discussion

The VTA in horizontal midbrain slices is identified and recognized as the area medial to the substantia nigra compacta and medial to terminal nucleus of the accessory optic tract. Additionally, a clear hyperpolarization-activated cation current (I_h_) emerges after hyperpolarizing the VTA DA neuron from -70 to -150 mv, in 10 mv step size, immediately after break-in was observed in each recorded neuron. I_h _was shown to be a reliable marker for VTA DA neurons [32-34]. A recent report has questioned the identification of VTA DA neurons using I_h _[35]. However, in previous studies [6,7,34,36] and in the present study, this criterion was sufficient to provide necessary identification.

The disruption between PFC and VTA was observed at the time the brain was removed from the skull. Once the brain has been removed from the skull, the area immediately caudal to PFC has been observed to be cut. This indicates the PFC has completely lost its connection with the rest of the brain.

Previous studies had reported that single nicotine injection could enhance the peak ratio of VTA DA neurons AMPA/NMDA receptors responses within 24 hours, even after nicotine metabolized [7]. Moreover, Mansvelder and McGehee reported brief nicotine application rapidly induced LTP and maintained for more than one hour in VTA slice [9]. Additionally, the positive correlation between glutamatergic synaptic enhancement and behavioral locomotion were well described [10]. *In vivo *experiments also showed that following nicotine application, dopamine release to NAc and the locomotion activity of rats both would peak within one hour [8]. These evidences led us to predict that, within only one hour after single nicotine injection, the postsynaptic response will be changed. Our hypotheses are supported by the results, since we found that even within one hour after a single nicotine injection, the peak ratio of AMPA/NMDA on VTA DA neurons could be enhanced. However, the peak ratio increase observed within one hour was lower in comparison to 24 hours after a single nicotine injection [7]. This suggests that, after one hour of a single systemic nicotine injection, not all subset of synapses were potentiated.

This finding was also supported by previous reports that the AMPA/NMDA ratio enhancement was observed two hours after a single cocaine injection [37] and observed in another study two hours after a single amphetamine injection [36].

The PFC is a key structure for executive functions of the brain [38,39] and has been shown to regulate the firing pattern of dopamine (DA) neurons in the VTA. Gao et al [28] stated that there is an indirect coupling between PFC and VTA. Thus, PFC stimulation increases burst firing in DA neurons, whereas PFC inactivation produces the opposite effect [40-44].

Glutamate transmission from PFC to VTA is important in controlling VTA DA neurons firing activities and animal behavior [11-15]. Treatments of most drugs of abuse has been reported to increase excitatory inputs to the midbrain, which is thought to contribute, impartially, to the enhancement of VTA DA neurons AMPA/NMDA ratio. All the evidences suggested it is a common mechanism of addiction through neural adaptation [6,7].

The disconnection of the functional pathway between PFC and VTA could significantly reduce the effects of drugs of addiction, including nicotine [9,28,45]. We recently have demonstrated in *in vivo *experiments that the responses of main VTA DA neurons to acute nicotine injection are greatly changed after the PFC transaction [23,24]. Based on these, in this study, we transected the PFC and examined the changes of AMPA/NMDA peak ratio of VTA DA neurons. Interestingly, without the intact input from PFC, the AMPA/NMDA ratio was still enhanced by nicotine injection.

Like LTP, AMPA/NMDA ratio alteration reflects the plasticity change in synapse. Normally these changes are caused by either increase in excitatory input or decrease in inhibitory input. In VTA, DA neurons receive excitatory inputs from PFC and the inhibitory inputs from GABAergic interneuron in VTA and/or NAc, which also should have functional coupling with PFC. The openings of GluR were changed after nicotine treatments, via the regulation from PFC to VTA DA neurons, that induced EPSCs. After PFC transection, the signals induced by nicotine could not be transferred from PFC to VTA adequately, it may be the result of the different alteration to GluR on VTA DA neurons. Measurement of AMPA/NMDA peak ratio only takes into account the maximum GluR response. However, the AMPA and NMDA curves represent the whole GluR response with respect to time. Therefore, we estimated the AMPA/NMDA area ratio and KL divergence to better understand the dynamics of AMPA and NMDA signals since they took into consideration the whole current response rather than just the peak response. With these two methods, we found that there is statistical significance between PFC intact and PFC transected rats with saline treatments as seen in Figures 4C and 5C. This is not observed when measuring the AMPA/NMDA peak ratio. One possible reason for the observed differences may be due to the loss of glutamatergic inputs from PFC, which is one of the most important factors that contribute to the ratio level [46].

The use of traditional analysis method of AMPA/NMDA peak ratio suggests the PFC is not a "must" area and the ratio enhancement could occur locally in VTA. Previous studies showed that *in vitro *exposure of VTA slices to amphetamine did not enhance AMPA/NMDA ratio [36]. However, local injection of amphetamine to the VTA *in vivo *triggered sensitization [47,48]. This suggests that the enhancement effects should be triggered in VTA and need other areas to provide functional feedback, increase the excitatory input and/or reduce the inhibitory input.

However, the use of different analysis methods based on AMPA/NMDA area ratio and KL divergence, show that PFC is suggested to play an important role in affecting the VTA DA neurons. These two methods took into account the dynamics of AMPA and NMDA signals of the complete response and not just the maximum, making them more suitable in understanding the effects caused by nicotine and PFC transection.

## Conclusions

We demonstrated that the ratio of AMPA/NMDA responses of VTA DA neuron could be enhanced by single nicotine injection even within one hour, and AMPA/NMDA area ratio and the KL divergence analysis method are able to provide a more complete understanding of AMPA and NMDA responses, and may be better fit for the analysis of other neurological signals.

## Competing interests

The authors declare that they have no competing interests.

## Authors' contributions

TC performed experiments, the data analysis, and helped to write the manuscript, DZ helped to write the manuscript, AD contributed to the data analysis and helped to write the manuscript, KK helped with the data analysis, YMA helped with the experiments, helped to interpret the data and write the paper. MA oversaw the data collection, the data analysis, helped to interpret the results and write the manuscript. All authors read and approved the final manuscript.
